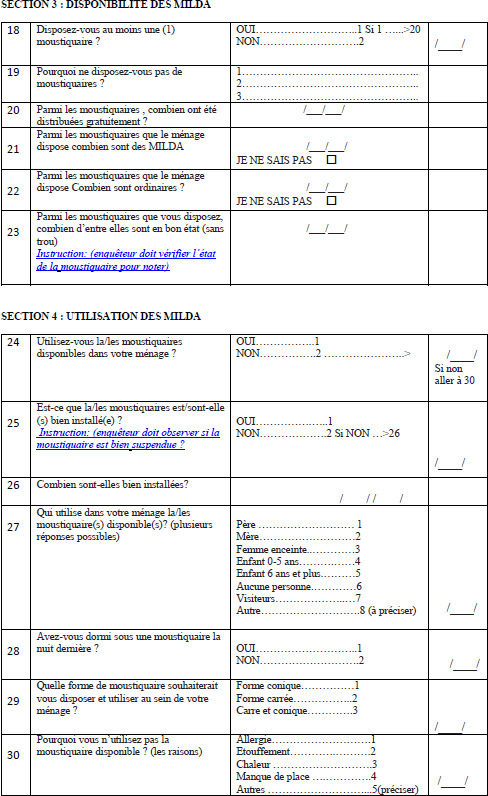# Étude comparative de la possession et de l’utilisation de moustiquaires imprégnées d’insecticide à longue durée d’action dans les régions de Man et Korhogo (Côte d’Ivoire)

**DOI:** 10.48327/mtsi.v6i1.2026.743

**Published:** 2026-03-25

**Authors:** KonanFabrice ASSOUHO, Florence FOURNET, Konan Rodolphe Mardoché AZONGNIBO, Akré Maurice ADJA

**Affiliations:** 1 UFR Agriculture, ressources halieutiques et agro-industries, Université de San Pedro, Côte d’Ivoire; 2 Institut Pierre Richet/Institut national de santé publique (IPR/INSP), Côte d’Ivoire; 3 MIVEGEC (Université de Montpellier, IRD, CNRS), 911 avenue Agropolis, BP 64501, 34394 Montpellier Cedex 5, France; 4 Institut de géographie tropicale (IGT), Université Félix Houphouët-Boigny (UFHB), Côte d’Ivoire; 5 UFR Biosciences, Université Félix Houphouët-Boigny, Côte d’Ivoire

**Keywords:** Paludisme, MILDA, Possession, Utilisation, Logokaha, Camp Sea, Kaforo, Kassiapleu, Man, Korhogo, Côte d’Ivoire, Afrique subsaharienne, LLIN, Ownership,Use, Logokaha, Camp Sea, Kaforo, Kassiapleu, Man, Korhogo, Côte d’Ivoire, Sub-Saharan Africa

## Abstract

**Justification:**

Le paludisme demeure un problème majeur de santé publique en Afrique subsaharienne. Pour prévenir la transmission de cette affection, des moustiquaires imprégnées d’insecticides à longue durée d’action (MILDA) ont été périodiquement distribuées en Côte d’Ivoire. La présente étude a pour objectif de déterminer la disponibilité et le taux d’utilisation des MILDA dans deux sites sentinelles du Programme national de lutte contre le paludisme (PNLP). Un site sentinelle est une localité sélectionnée de manière stratégique par le PNLP afin d’assurer une surveillance épidémiologique, parasitologique et entomologique continue et standardisée du paludisme.

**Matériel et méthodes:**

Une enquête transversale descriptive a été conduite entre janvier et mars 2019 auprès de 200 chefs de ménage des localités urbaines (Camp Sea et Logokaha) et rurales (Kassiapleu et Kaforo) de Man et Korhogo, deux sites sentinelles présentant un contexte épidémiologique différent pour le paludisme. Les données ont été collectées sur la base de questionnaires administrés aux chefs de ménage ou leurs répondants à leur domicile. Ils ont permis de recueillir des informations relatives aux caractéristiques sociodémographiques du chef de ménage, à sa perception et à sa connaissance du risque de paludisme, à la possession et à l’utilisation de MILDA au sein de son ménage.

**Résultats:**

Les ménages associaient la transmission du paludisme à des piqûres de moustiques dans au moins 70 % des cas. En outre, 87 % des enquêtés ont déclaré posséder au moins une MILDA et 84 % affirmaient les avoir utilisées la nuit précédente. Ce taux d’utilisation est supérieur à celui de 80 % préconisé par l’Organisation mondiale de la santé.

**Conclusion:**

Il ressort de ce travail que les populations enquêtées ont une bonne connaissance du paludisme et utilisent les MILDA pour se protéger, quel que soit le contexte de transmission. Cependant, certains résultats suggèrent que des mesures pourraient être prises par les autorités sanitaires pour améliorer l’utilisation des MILDA par les populations.

## Abstract (en)

**Rationale:**

Malaria remains a significant public health concern in sub-Saharan Africa. To prevent the transmission of the disease, long-lasting insecticide-treated mosquito nets (LLINs) have been periodically distributed in Côte d’Ivoire. This study aims to determine the availability and utilization rate of LLINs in two National Malaria Control Program (NMCP) sentinel sites. A sentinel site is a location selected by the NMCP to ensure continuous, standardized malaria epidemiological, parasitological, and entomological surveillance.

**Materials and methods:**

A descriptive, cross-sectional survey was conducted from January to March of 2019. Two hundred heads of households were surveyed in urban (Camp Sea and Logokaha) and rural (Kassiapleu and Kaforo) areas in Man and Korhogo. These two sites have different malaria epidemiological contexts. Data were collected using questionnaires administered to household heads or their representatives at home. The questionnaires gathered information on the household heads’ sociodemographic characteristics, their perceptions and knowledge of malaria risk, and their possession of and experience with using LLINs within their households.

**Results:**

In at least 70% of cases, households associated malaria transmission with mosquito bites. Additionally, 87% of respondents reported owning at least one LLIN, and 84% said they had used it the previous night. This usage rate exceeds the 80% recommended by the World Health Organization.

**Conclusion:**

This study shows that the surveyed populations have a good understanding of LLINs to protect themselves, regardless of the transmission context. However, some results suggest that health authorities could take measures to improve LLIN use among these populations.

**Figure:**
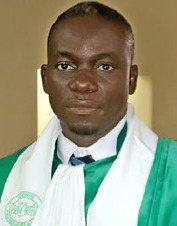

## Introduction

Le paludisme demeure d’actualité en Côte d’Ivoire. Des progrès significatifs ont été réalisés dans la lutte contre la maladie [8] : le nombre de décès dus au paludisme est passé de 3 222 en 2017 à 1 316 en 2020, soit un taux de létalité en baisse d’environ 50 %, et l’incidence nationale du paludisme chez les enfants de moins de cinq ans a diminué de 26,1 %. Il reste cependant l’une des premières causes de morbidité dans le pays. L’analyse des données de la surveillance entomologique a montré que sa transmission est permanente et hétérogène dans le pays [3]. Elle est assurée essentiellement par trois vecteurs : *Anopheles gambiae, An. funestus* et *An. nili [2,3].* Pour réduire le fardeau de cette maladie, le ministère de la Santé, de l’hygiène publique et de la couverture maladie universelle de Côte d’Ivoire recommande l’utilisation des combinaisons thérapeutiques à base de dérivés d’artémisinine (CTA) dans le traitement du paludisme simple, et l’utilisation de moustiquaires imprégnées d’insecticide à longue durée d’action (MILDA) pour la prévention de cette affection.

De 2014 à 2015, 13 millions de MILDA ont été distribuées aux populations ivoiriennes, auxquelles s’y ajoutent environ 15 millions en 2017 [4]. En outre, le Programme national de lutte contre le paludisme (PNLP) met à la disposition des centres de santé des moustiquaires pour garantir leur disponibilité permanente et gratuite pour les populations les plus vulnérables, c’est-à-dire les femmes enceintes et les enfants de moins de 5 ans. Pour mener à bien la distribution de MILDA, un dénombrement de ménages a été réalisé. Selon les recommandations de l’OMS, il faut distribuer une MILDA pour deux personnes [13,20]. Cela a permis d’augmenter le taux de couverture de MILDA de 66 % à 95 % entre 2015 et 2017. Cependant, peu d’informations existent sur leur utilisation. Les études montrent que le manque de connaissance d’un objet technique peut influencer les pratiques de son utilisation [7]. Une meilleure perception des signes cliniques d’une maladie, de son mode de transmission, de sa prévention et de son traitement est le gage de la réussite d’un programme de lutte [11,19].

Dans le cadre de la lutte contre le paludisme, un site sentinelle est une localité sélectionnée de manière stratégique par le PNLP afin d’assurer une surveillance épidémiologique, parasitologique et entomologique continue et standardisée du paludisme. Ces sites permettent de suivre les tendances spatio-temporelles de la transmission, d’évaluer l’impact des interventions (MILDA, traitement préventif intermittent, prise en charge), et de détecter précocement toute modification de la dynamique de la maladie ou de la résistance des vecteurs et des parasites [21]. Ils suivent également des indicateurs clés : nombre de cas testés, taux de positivité, cas confirmés, prise en charge thérapeutique, décès, etc. L’objectif est d’utiliser ces données pour faciliter la prise de décision du PNLP, l’allocation de ressources, l’identification de zones à haut risque et l’évaluation de l’impact des interventions systématiques, avec des rapports trimestriels [1]. Le but de cette étude, conduite à l’échelle des ménages de deux sites sentinelles du PNLP de la Côte d’Ivoire, est de déterminer la disponibilité des MILDA dans les unités familiales, leur degré d’utilisation ainsi que les freins à leur recours.

## Matériel et méthodes

### Sites d’étude

**Figure 1:**
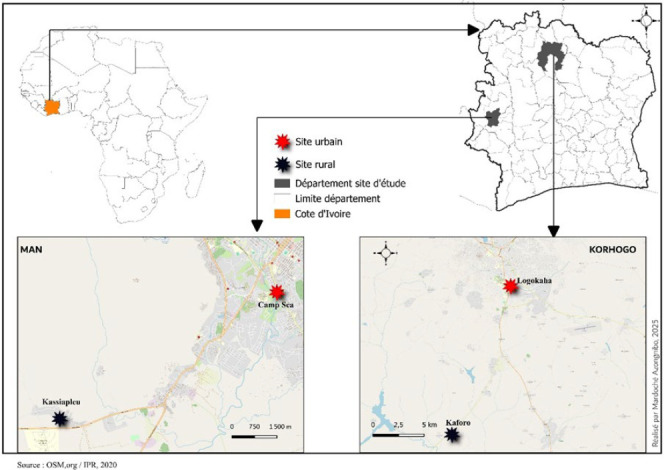
Présentation des sites d’étude de Man et de Korhogo

Deux départements, sites sentinelles suivis par le PNLP, ont été choisis pour cette étude [9,14] : le site de Korhogo, situé au nord du pays en zone de savane, et celui de Man à l’ouest en zone forestière (Fig. 1).

La région de Korhogo est sous l’influence d’un climat soudanais avec une alternance de deux saisons. La saison sèche commence en novembre et se termine en avril. La saison des pluies s’étend de mai à octobre avec des pluviométries maximales en juillet et août. La pluviométrie annuelle oscille entre 1 100 et 1 600 mm [5]. Les températures moyennes varient entre 24 °C et 33 °C. La végétation, comme celle de toute la région, est composée de savane arborée. Deux localisations ont été retenues.

Logokaha est un quartier périurbain, situé au sud-ouest de la ville de Korhogo. Un bas-fond rizicole le sépare de son voisin Premafolo à l’est. C’est un espace qui associe traits urbains et ruraux. Des bâtiments bas en dur remplacent progressivement des habitations en terre en voie de disparition. Les habitants sont globalement chrétiens, musulmans ou animistes (Poro).

Kaforo est village situé sur l’axe Korhogo-Guimbé à 20 km de la ville de Korhogo. Le village est construit de manière générale en semi-dur, des briques en terre crue revêtues de ciment. C’est un site électrifié avec des populations de religions musulmane et animiste.

La région de Man est connue pour son relief accidenté avec des sommets pouvant atteindre 1 200 m d’altitude [10]. Le climat y est très humide et caractérisé par deux saisons : une longue saison pluvieuse (8 mois entre mars et octobre) avec des précipitations annuelles comprises entre 1 600 à 2 500 mm et une courte saison sèche (4 mois entre novembre et février). Les températures moyennes varient entre 29 °C et 35 °C. La végétation comprend des forêts (forêts denses semi-décidues, forêts claires), et des savanes (savanes boisées, savanes arborées, savanes arbustives). Deux localisations ont également été retenues.

Camp Sea, espace urbain au centre de la ville de Man est un quartier résidentiel où l’administration et les logements des administrateurs dominent. Le quartier est construit totalement en dur avec des maisons basses. Les habitants sont à majorité chrétiens.

Kassiapleu est un village situé sur l’axe Man-Danané à 12 km de la ville de Man. Il comprend des bâtiments en dur et d’autres en terre. Les habitants sont en majorité chrétiens et animistes.

La présence de l’Université polytechnique de Man a profondément modifié l’environnement du site. De nombreuses habitations ont été refaites en dur. À l’image des quartiers urbains, c’est un village électrifié avec de l’eau courante.

Ces deux sites ont été choisis comme zones d’étude du fait de la forte agressivité anophélienne et du niveau de transmission du paludisme qui ont été relevés. Les taux d’agressivité des anophèles vecteurs étaient compris entre 26 et 52 piqûres par personne et par nuit. Les travaux d’Adja *et al.* menés de juin 2015 à mars 2016 ont montré que le taux d’inoculation entomologique variait entre 95 et 483 piqûres infectantes par personne et par an. Cette même étude avait mis l’accent sur la différence qui existait entre les deux sites sentinelles [2]. Le district sanitaire de Man avait un taux d’inoculation entomologique plus élevé que celui de Korhogo, traduisant un niveau de transmission élevé à Man et plus faible dans la localité de Korhogo. L’hypothèse qui guide cette contribution scientifique est que les populations de Man devraient davantage recourir aux MILDA que celles de Korhogo.

Chaque site sentinelle inclut un site urbain et un site rural. Les quartiers de Logokaha et Camp Sea correspondent aux zones urbaines à Korhogo et à Man respectivement. Les villages de Kaforo et Kassiapleu sont dans les zones rurales de Korhogo et Man respectivement (Fig. 1). Tous les sites sentinelles ont bénéficié des campagnes de distribution gratuite de MILDA en 2015 et en 2017 et continuent d’en bénéficier tous les deux ans par le PNLP de Côte d’Ivoire.

Les données ont été collectées à travers une enquête transversale sur la base de questionnaires administrés de janvier à mars 2019 à des chefs de ménage ou à leurs répondants à leur domicile (Annexe). Selon certaines études, la saison sèche est la période la plus propice pour évaluer l’utilisation d’un outil comme les MILDA car la menace perçue du paludisme est alors relativement faible [11]. Le questionnaire a permis de recueillir des informations relatives aux caractéristiques sociodémographiques du chef de ménage (âge, genre, niveau d’instruction, situation matrimoniale), à sa perception et à sa connaissance du risque de paludisme, à la possession et à l’utilisation de MILDA au sein de son ménage. La collecte des données a été faite par une équipe de deux personnes aidées par des guides locaux, des personnes reconnues dans leur environnement (quartier ou village). Ils sont au moins bilingues, comprennent leurs langues maternelles et le français. Les questionnaires ont été préparés en français et traduits en langue locale en cas de besoin pendant les entretiens. Des consentements éclairés ont été obtenus des chefs de ménage. L’anonymat des informations a été maintenu pendant toute l’étude.

Le choix des ménages à enquêter a été fait à partir d’un échantillonnage spatial opéré avec le logiciel QGIS d’information géographique version 3.34. Le bâti des différents sites a été numérisé à l’aide d’une imagerie satellite (Google satellite) et de JOSM, l’outil d’édition de données d’*OpenStreet-Map* (OSM), puis transféré dans QGIS. Le choix de 50 ménages par site suit une approche d’échantillonnage pragmatique et comparative couramment utilisée dans les enquêtes CAP et les enquêtes opérationnelles sur les ménages soutenues par l’Organisation mondiale de la santé (OMS). Ces modèles privilégient une représentation équilibrée entre les sites afin d’améliorer la comparabilité et la faisabilité plutôt que les tests d’hypothèses formels ou le calcul de la puissance statistique [6,10,19]. L’outil « *sampling* » de QGIS a été utilisé pour identifier 50 points dans chaque site, chaque point correspondant à un ménage à enquêter. Les coordonnées géographiques des bâtiments sélectionnés ont été enregistrées sous un format GPX (*GPS eXchange Format*) et transférées dans *OsmAnd* (application de navigation compatible avec les smartphones). L’enquêteur pouvait alors visualiser sur l’écran de sa tablette la position des ménages qu’il devait enquêter.

Les participants devaient être âgés d’au moins 18 ans. Lorsque le chef de ménage était absent, son épouse pouvait le remplacer. En cas de refus de participation ou d’inéligibilité du ménage tiré au sort, le ménage situé immédiatement à gauche était sélectionné comme substitut, ou celui de droite si le premier ne remplissait pas les critères requis. Le taux de refus observé a été de 4 %.

Les données recueillies ont été saisies dans le logiciel Epi Info 7.2.6 pour constituer la base de données, enregistrée ensuite et transférée dans QGIS. Le taux de couverture en MILDA a été défini comme étant le nombre de ménages possédant au moins une moustiquaire sur le nombre total de ménages enquêtés par site d’étude. Le taux d’utilisation correspondait au nombre de ménages qui déclarait avoir dormi sous une moustiquaire la nuit précédant l’enquête sur le nombre total de ménages enquêtés par site d’étude.

Le test de chi2 a permis de comparer ces taux entre les différents sites à travers le logiciel R 4.1.2 avec un intervalle de confiance de 95 %.

L’analyse spatiale de la distribution des ménages en fonction de l’utilisation habituelle de MILDA a été réalisée avec le logiciel GeoDa 1.22 en calculant l’indice de Moran (I de Moran), indicateur de la structure spatiale des évènements géographiques, qui a permis d’analyser la structure spatiale de la répartition des possesseurs de MILDA dans nos deux espaces d’étude. L’indice est compris entre -1 et 1. S’il est > 0, il y a une autocorrélation spatiale positive : les valeurs similaires (hautes ou basses) sont regroupées dans l’espace (agrégation). Cela exprime qu’il y a une agrégation des utilisateurs. S’il est égal à 0, il n’y a aucune autocorrélation spatiale (la distribution du phénomène est aléatoire). S’il est < 0, l’autocorrélation spatiale est négative : les valeurs dissemblables sont regroupées (dispersion spatiale du phénomène étudié). Ce résultat signifie qu’un utilisateur de MILDA est entouré d’un non-utilisateur de MILDA [10,20].

## Résultats

Au total, 200 chefs de ménage ou leurs représentants ont été interrogés (Tableau I). La taille moyenne des ménages était de 5,5 personnes. Le sex-ratio le plus élevé a été observé à Kaforo (1,2) avec une taille moyenne de ménages la plus faible (4,7). Camp Sea a notifié le plus faible sex-ratio (0,5) et une taille moyenne de ménages la plus élevée (6,8). Les sites de Kassiapleu et Logokaha avaient un sex-ratio autour de 0,80. Camp Sea présentait le nombre moyen de MILDA le plus élevé (3,0) et Kaforo le nombre le plus faible (1,2), sans que la différence soit significative.

En moyenne, seulement la moitié des chefs de ménage (50 %) a été scolarisée et c’est dans la zone de Man que le taux de scolarisation était le plus élevé (66 %) par rapport à Korhogo (34 %) (p < 0,0001). Il y avait davantage de musulmans scolarisés en milieu urbain qu’en milieu rural. Plus de 32 % des ménages scolarisés en milieu urbain étaient de religion musulmane, aussi bien à Camp Sea qu’à Logokaha. Kaforo a notifié le plus faible taux de chefs de ménage scolarisés de religion musulmane (7 %). Les répondants ou chefs de ménages étaient majoritairement de religion chrétienne (Camps Sea : 70 %; Kassiapleu : 54 %; Logokaha : 44 %) sauf à Kaforo où les animistes et musulmans dominent (48 % et 44 %). Le taux de scolarisation des femmes était plus élevé à Camp Séa (44 %) et plus faible à Kaforo (4 %) (Tableau I). De manière générale, 81,5 % des enquêtés déclaraient que le paludisme est transmis à travers la piqûre de moustiques. Kaforo est le site où cette proportion était la plus faible (70 %), sans différence statistique cependant avec les autres sites (p = 0,60) (Tableau II).

**Tableau I:** Caractéristiques sociales des enquêtés

	Man		Korhogo	
	Camp Sea*	Kassiapleu**	Logokaha*	Kaforo**
Nombre de bâtiments numérisés	704	519	882	249
Taille moyenne des ménages enquêtés (personnes/ménage)	6,8	5,3	5,3	4,7
Sex-ratio des ménages	0,5	0,8	0,8	1,2
Niveau d’éducation du chef de ménage				
non scolarisé (%)	24	44	50	82
scolarisé (%)	76	56	50	18
Religions (%)				
animiste	4	32	22	48
chrétien	70	54	44	8
musulman	26	14	34	44
Taux de scolarisation des chefs de ménage femme	44	18	26	4

* : site urbain; ** : site rural

**Tableau II:** Profil d’utilisation des MILDA et déterminants de leur non-utilisation selon le milieu de résidence

	Man		Korhogo	
	Camp Sea*	Kassiapleu**	Logokaha*	Kaforo**
Nombre de MILDA par ménage	3	2,5	2,3	1,2
Catégorie d’utilisateur de MILDA (%)				
enfant de 0 à 5 ans	6	0	32	20
enfant de plus de 6 ans	10	6	8	2
femme enceinte	0	0	2	0
mère	2	4	10	20
père	2	8	8	24
autres	60	78	26	6
Signes du paludisme (%)				
fièvre	42	42	30	12
maux de tête	28	12	18	10
perte d’appétit	10	8	6	4
refroidissement/frissons	6	10	4	2
vomissements	4	4	24	50
autres	10	24	26	22
Modes de transmission (%)				
piqûre de moustiques	86	90	80	70
eau/environnement/lieu sale	10	2	2	4
autres	4	8	18	26
Protection contre le paludisme (%)				
moustiquaires	76	86	72	82
serpentins insecticides	4	0	0	4
assainissement du milieu	6	4	18	2
grillages aux portes et fenêtres	2	2	0	0
bombes insecticides	6	0	0	0
autres	6	4	10	14
Possession d’au moins une MILDA dans le ménage (%)	90	98	90	70
Utilisation la nuit précédente (%)	68	86	82	70
Motifs de non-utilisation de MILDA (%)				
chaleur	38,5	50	30,8	0
étouffement	7,7	16,7	23,1	0
manque de place	7,7	0	0	0
allergie	0	0	7,7	0
ne sait pas	46,2	16,7	38,5	30

* : site urbain; ** : site rural

La fièvre était le symptôme le plus fréquemment rapporté lors d’épisodes attribués au paludisme (42 % à Camp Sea et à Kassiapleu, 30 % à Logokaha), ce qui est attendu pour une maladie fébrile, mais non spécifique en l’absence de confirmation parasitologique. À Kaforo, ce sont les vomissements qui étaient indiqués en premier par la moitié des enquêtés (Tableau II).

**Figure 2:**
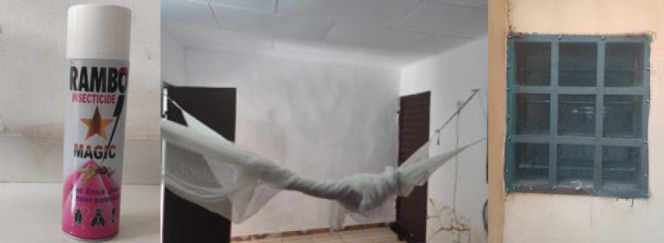
Spray d’insecticide. MILDA installée Grillage installé sur une fenêtre

Le taux moyen de possession de MILDA dans les localités échantillonnées était de 87 %, sans différence significative entre les sites (p = 0,43). Kaforo se distingue par une possession plus faible (70 %) mais une utilisation maximale (Tableau II). Kassiapleu a notifié la plus forte proportion de possession de MILDA (98 %). D’autres outils de prévention étaient utilisés (serpentins insecticides, assainissement du milieu, grillages aux portes et fenêtres). Les ménages de Camp Sea sont les seuls à utiliser les sprays insecticides contre le paludisme (6 %) (Fig. 2). Au total, les MILDA avaient été utilisées la veille de l’enquête dans 84 % des ménages enquêtés. Le site de Kaforo affiche la plus grande proportion d’utilisation avec une différence significative avec les autres sites (p = 0,10).

Les utilisateurs de MILDA étaient généralement les enfants de 0 à 5 ans à Logokaha (32 %), les mères, pères et les enfants de 0 à 5 ans à Kaforo, respectivement 24 %, 20 % et 20 %. À Kassiapleu et à Camp Sea, la catégorie « autres » (enfants d’un proche ou étranger) dominait la proportion des utilisateurs (78 % et 60 %) (Tableau II).

**Figure 3:**
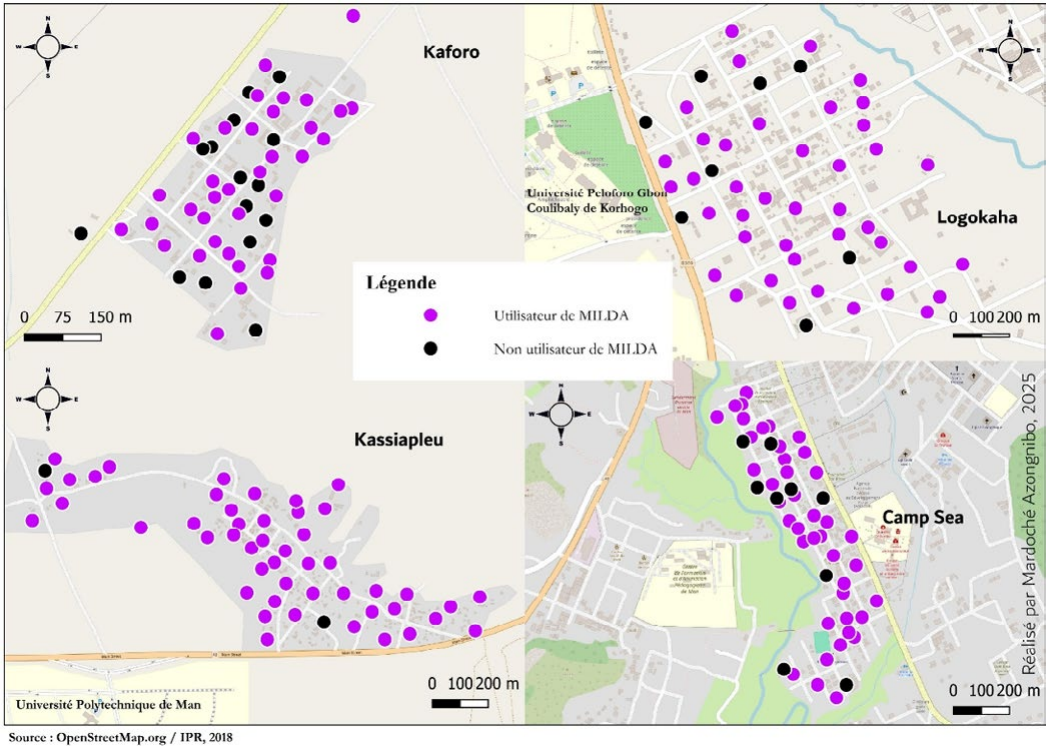
Répartition des utilisateurs de MILDA dans chaque zone enquêtée

Les raisons de la non-utilisation habituelle des MILDA variaient d’un site à un autre. Dans l’ensemble, la chaleur et l’étouffement étaient les principales raisons avec respectivement 36 % et 15 % des déclarations . Cependant une grande proportion des enquêtés (36 %) n’a pas su expliquer les motifs de la non-utilisation de MILDA. La distribution spatiale des utilisateurs de MILDA semble assez homogène dans tous les sites. En milieu rural, nous avons observé que les non-utilisateurs de MILDA étaient plus nombreux à Kaforo (15/50), qu’à Kassiapleu (2/50) (Fig. 3). En milieu urbain (Logokaha et Camp Sea), les non-utilisateurs étaient respectivement de 8/50 et 9/50. L’analyse a mis en évidence une autocorrélation spatiale très faible et négative. Le I de Moran était respectivement de -0,015 à Logokaha, -0,026 à Kaforo, -0,058 à Camp Sea et -0,032 à Kassiapleu. Ces indices révèlent une absence de structure spatiale forte dans ces différents sites. Les résultats suggèrent que dans ces localités, il n’y a pas de regroupements spatiaux significatifs, mais plutôt une certaine dispersion ou alternance d’utilisateurs et de non-utilisateurs de MILDA dans l’espace proche.

## Discussion

Nous avons noté un bon niveau de connaissance du paludisme et de ses modalités de transmission. Plus de 70 % des enquêtés identifiaient les moustiques comme le mode de transmission de la maladie. Ce résultat pourrait être associé à un niveau de scolarisation élevé dans l’ensemble des sites de cette étude. Une étude menée dans la région ouest du Cameroun a montré que le niveau de connaissances élevé des populations sur le mode de transmission du paludisme était lié au fait que les localités comptaient parmi les plus scolarisées de la région [17]. Ces résultats ont aussi été observés au Gabon [15]. Il est important de noter qu’une partie de la population continue de citer l’eau sale comme cause du paludisme, d’où l’intérêt de poursuivre les sensibilisations en vue d’améliorer les connaissances.

Notre étude a montré que le taux de possession de MILDA dans les sites sentinelles de Korhogo et de Man était élevé (87 %) et conforme aux objectifs des campagnes de distribution de masse menées par le PNLP. Ce taux de possession est plus fort que celui observé dans des villages du département de Tiébissou (Côte d’Ivoire) qui était de l’ordre de 65 % [12]. Cette situation pourrait être due au fait que cette dernière étude a été conduite uniquement en milieu rural et hors des sites sentinelles.

Dans les sites sentinelles, les populations pourraient être davantage sensibilisées sur le paludisme du fait de la présence fréquente des équipes du PNLP.

Cependant, si on considère la taille moyenne des ménages (5,5 personnes) enquêtés et le nombre moyen de MILDA disponibles par foyer (1,2 en moyenne), nos résultats montrent une couverture incomplète et sa non-conformité aux recommandations de l’OMS (une MILDA pour deux personnes) [9].

Le taux d’utilisation à la veille de l’enquête des MILDA étaient compris entre 68 % et 86 % pour les ménages déclarant en posséder. Des différences entre taux de possession et taux d’utilisation ont été observées dans plusieurs études qui ont souligné que la possession ne garantissait pas toujours l’utilisation effective des moustiquaires [23]. Le taux d’utilisation pour les enfants est inférieur à 35 %. Nos résultats sont différents de ceux de Ladu *et al*. [15]. Cette différence pourrait être due à la source de données : nos données sont des données primaires et celles analysées par Ladu *et al.* sont issues d’enquêtes de démographie et de santé 2011-2012.

Les taux de possession et d’utilisation des MILDA diffèrent légèrement entre les deux zones d’étude. La possession est plus élevée à Man qu’à Korhogo (94 % *vs* 80 %), tandis que l’utilisation effective est plus forte à Korhogo qu’à Man (91 % *vs* 77 %). Ces différences sont statistiquement significatives au seuil de 5 %, mais d’amplitude modérée.

Le faible niveau de transmission observé à Korhogo par rapport à Man par Adja *et al.* [2] pourrait être en partie dû au fort taux d’utilisation effectif des MILDA par les ménages, contrairement aux populations de Man qui en possèdent davantage mais qui les utilisent moins.

Nos résultats sont identiques à ceux observés au Cameroun dans trois districts sanitaires [6]. Dans des districts sanitaires avec des niveaux de prévalence palustre différents, il apparaît que les populations avaient un taux de possession et d’utilisation différents d’un district à un autre. L’une des principales raisons évoquées pour la non-utilisation des MILDA à Kaforo est l’inconfort lié à la chaleur, ce qui rejoint les conclusions d’études antérieures soulignant que les barrières comportementales et contextuelles restent des freins majeurs à leur utilisation optimale [24]. Selon ces travaux, les raisons de la non-utilisation des MILDA dans des pays endémiques pour le paludisme sont les facteurs humains (croyances et pratiques socioculturelles), les facteurs liés aux moustiquaires, la structure du logement et l’accès aux moustiquaires. Les facteurs socioéconomiques et éducatifs influencent aussi la possession et l’utilisation des MILDA. Plusieurs études ont montré que le niveau d’éducation était corrélé positivement avec l’adoption des comportements préventifs contre le paludisme [7]. Le taux de scolarisation plus élevé à Man (66 %) qu’à Korhogo (34 %) pourrait y expliquer la plus importante possession de MILDA. Néanmoins, une proportion importante des enquêtés à Camp Sea (46 %) et à Logokaha (38 %) n’a pas pu expliquer les raisons de la non-utilisation des MILDA. Cette situation a également été observée au niveau du district sanitaire de Dimbokro, où 19,8 % des ménages n’ont pas pu expliquer pourquoi ils ne les utilisaient pas [25].

Le recours à d’autres méthodes de prévention (serpentins et sprays insecticides, grillages de fenêtres), notamment à Camp Sea, montre que les populations combinent plusieurs stratégies. Cette situation pourrait être associée au comportement des vecteurs du paludisme. En effet, les principaux vecteurs du paludisme en Côte d’Ivoire (*An. gambiae* et *An. funestus*) piquent autant à l’intérieur qu’à l’extérieur des habitations et à des périodes de la nuit où les populations ne sont pas toujours couchées [3,12,16,18].

Les faibles taux de répondants citant l’assainissement comme moyen de lutte (de 2 % à 18 % dans nos différents sites) suggèrent que les mesures de gestion environnementale (drainage, suppression des eaux stagnantes, hygiène du cadre de vie, gestion des déchets, etc.) sont moins reconnues comme stratégies antipaludiques que les mesures individuelles (moustiquaires, insecticides). Cette hiérarchie des réponses est cohérente avec de nombreuses enquêtes, dans lesquelles les pratiques « directes » (MILDA, pulvérisation, répulsifs) dominent la perception communautaire, tandis que l’assainissement apparaît comme une mesure diffuse, souvent considérée comme relevant davantage des collectivités, municipalités ou structures sanitaires que des ménages, et requérant une compréhension plus technique des gîtes larvaires et de l’écologie vectorielle [24].

Cette faible citation de l’assainissement peut également refléter une disjonction entre connaissance et action. Des populations sachant que l’environnement joue un rôle dans la lutte contre le paludisme, peuvent ne pas l’identifier comme une « méthode de lutte » prioritaire. Le manque de moyens matériels ou organisationnels pour agir est aussi l’une des raisons. Des travaux sur la gestion des gîtes larvaires menés ont montré que, bien que des pratiques environnementales existent, leur appropriation communautaire dépend fortement des ressources, de la coordination collective et du soutien institutionnel [25].

La représentativité de nos sites pourrait être une limite. En effet, si le district sanitaire est composé d’espaces ruraux et urbains, un site par milieu peut être insuffisant pour représenter les différents espaces. L’absence d’informations élémentaires sur les MILDA (nombre, insecticide utilisé, etc.) dans nos différents sites d’étude est aussi l’une des limites à relever.

Bien que l’étude ait été menée après la distribution nationale de masse de MILDA, *il demeure difficile d’attribuer avec certitude une éventuelle réduction de la transmission du paludisme à la seule possession ou utilisation de ces moustiquaires.* Les comportements de possession et d’usage de MILDA observés chez les enquêtés devraient être associés à une évaluation de la prévalence de la maladie. Cela permettrait de mieux apprécier l’effet de l’acceptabilité et de l’utilisation de ce moyen de protection sur la maladie. La période de l’enquête peut influencer certains résultats. Même s’il est rapporté que la saison sèche est la meilleure période pour ce type d’étude, une étude sur toute l’année permettrait une meilleure appréciation de l’objet étudié.

## Conclusion

Les populations des sites sentinelles de Korhogo et de Man connaissent bien le mode de transmission du paludisme ainsi que ses signes. La MILDA est l’outil de prévention le plus utilisé par ces populations, en combinaison avec d’autres moyens de prévention comme l’usage de serpentins insecticides, de grillages et de rideaux de fenêtres. Il ressort de notre analyse une couverture incomplète, en non-conformité aux recommandations de l’OMS et du PNLP. Bien que les taux de possession et d’utilisation de MILDA apparaissent relativement conformes aux attentes du PNLP, ils semblent être impactés par certains freins (chaleur, étouffement) aussi bien pour l’accès aux MILDA que pour leur utilisation. Ces freins traduisent le besoin d’améliorer la compréhension des enjeux liés à la non-utilisation de ce moyen de prévention essentiel, et de poursuivre la sensibilisation des populations en l’adaptant au contexte local. Il semble également important de pouvoir conduire de telles enquêtes dans les autres sites sentinelles du PNLP.

## Remerciements

Nous remercions les autorités administratives et traditionnelles ainsi que les populations interviewées de Korhogo et de Man qui ont donné de leur temps pour participer à l’enquête.

## Déclaration de liens d’intérêt, financement et principes éthiques

Cette recherche n’a bénéficié d’aucun soutien financier et aucun lien d’intérêt n’a été déclaré. L’étude a reçu l’approbation des autorités sanitaires de chaque localité. L’autorisation d’enquête a été obtenue auprès des chefs de quartiers/village. De plus, un consentement verbal des enquêtés avait été obtenu au préalable avant de participer à l’étude.

## Contribution des auteurs et autrices

KFA, AMA, FF ont participé à l’élaboration du sujet et l’écriture du manuscrit. KFA et KRMA ont participé à l’enquête. KFA a conduit la saisie et l’analyse des données. KFA, KRMA, FF ont participé à la rédaction du manuscrit. KFA, KRMA, AMA et FF ont participé à la correction du manuscrit et tous les auteurs ont donné leur accord pour sa publication.

## Annexe : Fiche d’enquête sur les facteurs associés à l’utilisation des MILDA

**Figure:**
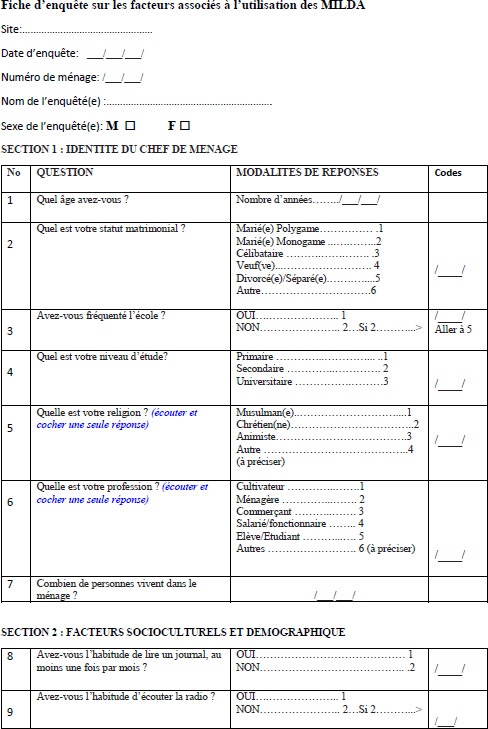

**Figure:**
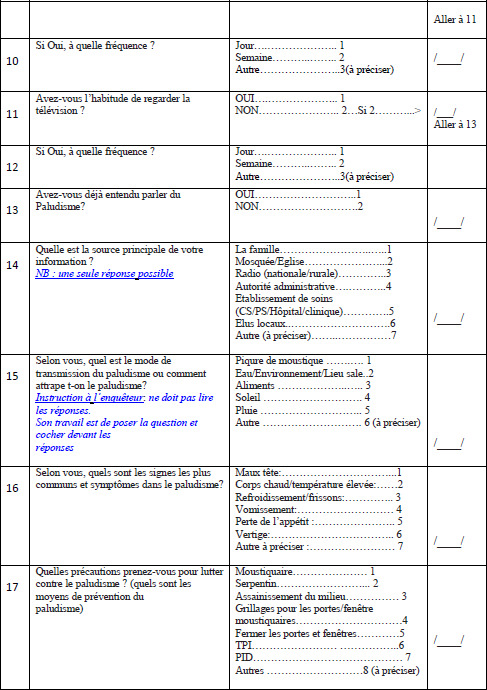

**Figure:**