# Supplementary material for: Comparative study of the ownership and use of long-lasting insecticide-treated mosquito nets in the Man and Korhogo regions (Côte d’Ivoire)
Source: Med Trop Sante Int. 2026 Mar 25;6(1):mtsi.v6i1.2026.743. [Article in French] doi: 10.48327/mtsi.v6i1.2026.743 (PMC13216900; doi:10.48327/mtsi.v6i1.2026.743)
Supplement: Related texts

# Research article: Comparative study of the ownership and use of long-lasting insecticide-treated mosquito nets in the Man and Korhogo regions (Côte d’Ivoire)

## Introduction

Malaria remains a pressing issue in Côte d’Ivoire. Significant progress has been made in the fight against the disease [8]: the number of malaria-related deaths fell from 3,222 in 2017 to 1,316 in 2020, representing a 50% decline in the case fatality rate. Additionally, the national incidence of malaria among children under five years of age decreased by 26.1%. Nevertheless, malaria remains one of the leading causes of morbidity in Côte d’Ivoire. Analysis of entomological surveillance data has shown that malaria transmission is permanent and heterogeneous throughout the country [3]. It is primarily transmitted by three vectors: *Anopheles gambiae*, *An. funestus*, and *An. nili* [2,3]. To reduce the disease’s burden, Côte d’Ivoire’s Ministry of Health, Public Hygiene, and Universal Health Coverage recommends artemisinin-based combination therapies (ACTs) for treating uncomplicated malaria and long-lasting insecticide-treated mosquito nets (LLINs) for preventing it.

From 2014 to 2015, 13 million LLINs were distributed to the Ivorian population, and an additional 15 million were distributed in 2017 [4]. Additionally, the National Malaria Control Programme (NMCP) provides health centers with mosquito nets to ensure they are permanently and freely available to vulnerable populations, including pregnant women and children under five years of age.

A household census was carried out to ensure the successful distribution of LLINs. According to WHO recommendations, one LLIN should be distributed for every two people [13,20]. This increased LLIN coverage from 66% to 95% between 2015 and 2017. However, little information exists on their use. Studies show that a lack of knowledge about a technical object can influence how it is used [7]. Understanding the clinical signs of a disease, how it is transmitted, and how it can be prevented and treated is key to the success of a control program [11,19].

In the context of malaria control, a sentinel site is a location that is strategically selected by the NMCP to ensure continuous and standardized epidemiological, parasitological, and entomological surveillance of malaria. These sites enable the monitoring of spatial and temporal trends in transmission, the evaluation of the impact of interventions such as LLIN, intermittent preventive treatment, malaria attacks treatment, and the early detection of changes in disease dynamics or vector and parasite resistance [21]. Sentinel sites also monitor key indicators such as the number of cases tested, positivity rate, confirmed cases, therapeutic management, and deaths. These data are used to facilitate NMCP decision-making, resource allocation, identification of high-risk areas, and evaluation of the impact of systematic interventions. Quarterly reports are produced [1]. This study, conducted at the household level in two NMCP sentinel sites in Côte d’Ivoire, aims to determine the availability of LLIN in family units, the extent to which it is used, and the barriers to its use.

## Materials and methods

### Study sites

The study sites were two departments monitored by the NMCP [9,14]: the Korhogo site in the northern savanna zone and the Man site in the western forest zone (Fig. 1).

**Figure 1:** Overview of the study sites in Man and Korhogo

The Korhogo region has a Sudanese climate with two alternating seasons. The dry season begins in November and ends in April. The rainy season lasts from May to October, with maximum rainfall in July and August. Annual rainfall varies between 1,100 and 1,600 mm. Average temperatures range from 24°C to 33°C. Like the rest of the region, the vegetation consists of wooded savanna. Two locations were selected. Logokaha is a suburban neighborhood located southwest of the city of Korhogo. A low-lying rice field separates Logokaha from its eastern neighbor, Premafolo. The area combines urban and rural features. Low-rise concrete buildings are gradually replacing the disappearing mud houses. The inhabitants are primarily Christian, Muslim, or animist (Poro). Kaforo is a village located on the Korhogo-Guimbé road, 20 kilometers from Korhogo. The village is built primarily with semihard, raw earth bricks coated with cement. It is an electrified site with Muslim and Animist populations.

The Man region is known for its rugged terrain, with peaks reaching up to 1,200 meters (3,937 feet) above sea level [10]. The climate is humid with two seasons: a long rainy season (eight months, from

March to October) with annual rainfall between 1,600 and 2,500 mm, and a short dry season (four months, from November to February). Average temperatures range from 29°C to 35°C. The vegetation includes forests (dense, semi-deciduous, and open) and savannas (wooded, tree, and shrub). Two locations have also been selected. Camp Sea is a residential neighborhood dominated by government buildings and administrators’ housing in the center of the city of Man. The neighborhood is made up entirely of solidly constructed, low-rise houses. Most of the inhabitants are Christian. Kassiapleu is a village located 12 km from the city of Man on the Man-Danané road. It has solid buildings as well as buildings made of earth. Most of the inhabitants are Christian or animist. The presence of Man Polytechnic University has profoundly changed the area. Many homes have been rebuilt with solid construction. Like urban neighborhoods, Kassiapleu has electricity and running water.

These two sites were chosen as study areas due to the high levels of *Anopheles* aggression and malaria transmission observed there. The *Anopheles* vector aggressiveness rates ranged from 26 to 52 bites per person per night. Adja *et al.’*s work, conducted from June 2015 to March 2016, showed that the entomological inoculation rate varied from 95 to 483 infectious bites per person per year. The same study revealed differences between the two sentinel sites [2]. The Man health district had a higher entomological inoculation rate than the Korhogo district, reflecting a high level of transmission in Man and a lower level in Korhogo. This study hypothesizes that the population of Man should make greater use of LLINs than the population of Korhogo.

Each sentinel site includes an urban and a rural site. The Logokaha and Camp Sea neighborhoods correspond to the urban areas of Korhogo and Man, respectively. The villages of Kaforo and Kassiapleu are in the rural areas of Korhogo and Man, respectively (Fig. 1). All sentinel sites received free LLIN distributions in 2015 and 2017 and continue to receive them every two years through the NMCP of Côte d’Ivoire.

Data were collected through a cross-sectional survey based on questionnaires administered from January to March of 2019 to household heads or their representatives in their homes (Appendix). According to some studies, the dry season is the most appropriate time to evaluate the use of a tool such as LLINs because the perceived threat of malaria is relatively low at that time [11]. The questionnaire collected information on the socio-demographic characteristics of the head of household (e.g., age, gender, level of education, and marital status), their perception of and knowledge about the risk of malaria, and whether their household possesses and uses LLINs. Data collection was carried out by a team of two people assisted by local guides well known in their community (neighborhood or village). They were at least bilingual, understanding both their native languages and French. The questionnaires were prepared in French and translated into the local language during the interviews when necessary. Informed consent was obtained from the heads of households. The anonymity of the information was maintained throughout the study.

Households were selected for survey using spatial sampling with the geographic information system QGIS version 3.34 software. Buildings at various sites were digitized using satellite imagery (Google Satellite) and JOSM, the data editing tool from OpenStreetMap. The data was transferred to QGIS. Selecting 50 households per site follows the pragmatic and comparative sampling approach commonly used in KAP and operational household surveys supported by the World Health Organization (WHO). These models favor balanced representation across sites to improve comparability and feasibility rather than formal hypothesis testing or statistical power calculations [6,10,19]. The QGIS “sampling” tool was used to identify 50 points on each site; each point corresponds to a household to be surveyed. The geographic coordinates of the selected buildings were recorded in GPX (GPS Exchange Format) and transferred to OsmAnd, a navigation application compatible with smartphones. The interviewer could then view the locations of the households to be surveyed on their tablet screen.

Participants had to be at least 18 years old. If the head of the household was absent, his or her spouse could be interviewed instead. If a randomly selected household refused to participate or was ineligible, the household immediately to the left was selected as a substitute, or the household immediately to the right if the first did not meet the required criteria. The refusal rate was 4%.

The data were entered into Epi Info 7.2.6 software to create a database. This database was then saved and transferred to QGIS. LLIN coverage was defined as the number of households with at least one mosquito net divided by the total number of households surveyed per study site. The usage rate corresponded to the number of households that reported sleeping under a mosquito net the night before the survey, divided by the total number of households surveyed per study site.

A chi-square test was performed using R 4.1.2 software with a 95% confidence interval to compare these rates between the different sites.

A spatial analysis of the distribution of households according to their habitual use of LLINs was performed using GeoDa 1.22 software. This analysis involved calculating Moran’s index (Moran’s I), which is an indicator of the spatial structure of geographical events. This calculation made it possible to analyze the spatial structure of the distribution of LLINs owners in our two study areas. The index ranges from -1 to 1; if it is greater than 0, there is positive spatial autocorrelation, meaning similar values (high or low) are grouped together in space (aggregation). This indicates an aggregation of users. If the index is equal to 0, there is no spatial autocorrelation, meaning the distribution of the phenomenon is random. If the index is less than 0, there is negative spatial autocorrelation, meaning dissimilar values are grouped together, indicating spatial dispersion of the phenomenon. This result means that a LLIN user is surrounded by a non- LLIN user [10,20].

## Results

A total of 200 heads of households or their representatives were interviewed (Table I). The average household size was 5.5 people. The highest sex ratio was observed in Kaforo (1.2), which had the lowest average household size (4.7). Camp Sea reported the lowest sex ratio (0.5) and the highest average household size (6.8). The Kassiapleu and Logokaha sites had a sex ratio of around 0.80. Camp Sea had the highest average number of LLINs (3.0), and Kaforo had the lowest (1.2); however, the difference was not significant.

On average, only half (50%) of household heads had attended school. The highest school enrollment rate was in the Man area (66%), compared to 34% in Korhogo (p < 0.0001). More Muslims attended school in urban areas than in rural areas. In urban areas, more than 32% of households with school-aged children were Muslim in both Camp Sea and Logokaha. Kaforo reported the lowest rate of Muslim heads of households with an education (7%). Most respondents or heads of households were Christian (Camp Sea: 70%; Kassiapleu: 54%; Logokaha: 44%), except in Kaforo, where animists and Muslims predominated (48% and 44%, respectively). The rate of female schooling was highest in Camp Sea (44%) and lowest in Kaforo (4%) (Table I).

**Table I:** Demographic characteristics of respondents

	Man		Korhogo	
	Camp Sea*	Kassiapleu**	Logokaha*	Kaforo**
Nombre de bâtiments numérisés / Number of digitized buildings	704	519	882	249
Taille moyenne des ménages enquêtés (personnes/ménage) / Average household size (people per household)	6,8	5,3	5,3	4,7
Sex-ratio des ménages / Gender ratio of households	0,5	0,8	0,8	1,2
Niveau d’éducation du chef de ménage / Education level of the head of household				
>non scolarisé (%) / No schooling (%)	24	44	50	82
>scolarisé (%) / Schooled (%)	76	56	50	18
Religions (%) / Religions (%)				
>animiste / Animist	4	32	22	48
>chrétien / Christian	70	54	44	8
>musulman / Muslim	26	14	34	44
Taux de scolarisation des chefs de ménage femme / Enrollment rate of female heads of household	44	18	26	4

* : site urbain; ** : site rural *: urban area; **: rural area

Overall, 81.5% of respondents reported that malaria is transmitted through mosquito bites. Kaforo had the lowest proportion (70%), though this did not differ significantly from the other sites (p = 0.60) (Table IT).

Fever was the most frequently reported symptom during malaria-attributed episodes (42% in Camp Sea and Kassiapleu and 30% in Logokaha), which is expected for a febrile illness but not specific without parasitological confirmation. In Kaforo, vomiting was the first symptom reported by half of the respondents (Table II).

The average LLIN ownership rate in the sampled locations was 87%, with no significant difference between sites (p = 0.43). Kaforo had lower ownership (70%) but maximum use (Table II). Kassiapleu reported the highest proportion of LLIN ownership (98%). Other prevention tools were also used, including insecticide coils, environmental sanitation, and nets on doors and windows. Households in Camp Sea were the only ones to use insecticide sprays against malaria (6%) (Fig. 2). Overall, 84% of the surveyed households had used LLINs the day before the survey. The Kaforo site had the highest proportion of LLIN use, significantly higher than the other sites (p = 0.10).

**Table II:** Usage patterns of LLINs and factors influencing their non-use by residential area

	Man		Korhogo	
	Camp Sea*	Kassiapleu**	Logokaha*	Kaforo**
Nombre de MILDA par ménage / Number of LLINs per household	3	2,5	2,3	1,2
Catégorie d’utilisateur de MILDA (%) / LLIN user category (%)				
>enfant de 0 à 5 ans / children aged 0-5	6	0	32	20
>enfant de plus de 6 ans / children over 6	10	6	8	2
>femme enceinte / pregnant woman	0	0	2	0
>mère / mother	2	4	10	20
>père / father	2	8	8	24
>autres / others	60	78	26	6
Signes du paludisme (%) / Symptoms of malaria (%)				
>fièvre / fever	42	42	30	12
>maux de tête / headache	28	12	18	10
>perte d’appétit / loss of appetite	10	8	6	4
>refroidissement/frissons / chills	6	10	4	2
>vomissements / vomiting	4	4	24	50
>autres / others	10	24	26	22
Modes de transmission (%) / Modes of transmission (%)				
>piqûre de moustiques / mosquito bites	86	90	80	70
>eau/environnement/lieu sale / dirty water/environment/ location	10	2	2	4
>autres / others	4	8	18	26
Protection contre le paludisme (%) / Malaria protection (%)				
>moustiquaires / mosquito nets	76	86	72	82
>serpentins insecticides / insecticide-treated coils	4	0	0	4
>assainissement du milieu / environmental sanitation	6	4	18	2
>grillages aux portes et fenêtres / screens on doors and windows	2	2	0	0
>bombes insecticides / insecticide sprays	6	0	0	0
>autres / others	6	4	10	14
Possession d’au moins une MILDA dans le ménage (%) / Household ownership of at least one LLIN (%)	90	98	90	70
Utilisation la nuit précédente (%) / Use the previous night (%)	68	86	82	70
Motifs de non-utilisation de MILDA (%) / Reasons for not using LLINs (%)				
chaleur / heat	38,5	50	30,8	0
étouffement / stuffy	7,7	16,7	23,1	0
manque de place / lack of space	7,7	0	0	0
allergie / allergy	0	0	7,7	0
ne sait pas / don’t know	46,2	16,7	38,5	30

*: urban area; **: rural area

**Figure 2:** Insecticide spray. LLIN installed. Screen installed on a window

LLIN users were generally children aged 0 to 5 in Logokaha (32%), and mothers, fathers, and children aged 0 to 5 in Kaforo (24%, 20%, and 20%, respectively). In Kassiapleu and Camp Sea, the “other” category (children of relatives or strangers) made up the majority of users (78% and 60%, respectively) (Table II).

The reasons for not regularly using LLINs varied by site. Overall, heat and stuffiness were the main reasons, with 36% and 15% of respondents citing these reasons, respectively. However, a large proportion of respondents (36%) could not explain why they did not use LLINs.

The spatial distribution of LLIN users appears to be fairly homogeneous across all sites. In rural areas, there were more non-users in Kaforo (15/50) than in Kassiapleu (2/50) (Fig. 3). In urban areas (Logokaha and Camp Sea), 8 and 9 out of 50 respondents were non-users, respectively.

**Figure 3:** Distribution of LLIN users in each surveyed area

The analysis revealed very weak and negative spatial autocorrelation. Moran’s I was -0.015 in Logokaha, -0.026 in Kaforo, -0.058 in Camp Sea, and -0.032 in Kassiapleu. These indices reveal a lack of strong spatial structure at these sites. The results suggest that these localities have no significant spatial clusters of LLIN users and non-users, but rather a certain dispersion or alternation of them in the immediate vicinity.

## Discussion

We observed a high level of knowledge regarding malaria and its transmission. More than 70% of respondents identified mosquitoes as the means by which the disease is transmitted. This result may be associated with the high level of education in all sites included in the study. A study conducted in western Cameroon showed that the population’s high level of knowledge about malaria transmission was linked to the fact that the localities were among the most educated in the region [17]. Similar results were observed in Gabon [15].

It is important to note that some in the population continue to cite dirty water as a cause of malaria; therefore, awareness campaigns are important to improve knowledge.

Our study showed that the LLIN ownership rate in the Korhogo and Man sentinel sites was high (87%), aligning with the NMCP’s mass distribution campaign objectives. This ownership rate is higher than that observed in villages in the Tiébissou department of Côte d’Ivoire, which was around 65% [12]. This difference may be due to the fact that the latter study was conducted only in rural areas and outside of sentinel sites.

In sentinel sites, populations may be more aware of malaria due to the frequent presence of NMCP teams.

However, considering the average household size (5.5 people) and the average number of LLINs per household (1.2), our results show incomplete coverage and noncompliance with WHO recommendations (one LLIN for every two people) [9]. The rate of LLIN use ranged from 68% to 86% among households that reported owning a net. Several studies have observed differences between ownership and usage rates, highlighting that ownership does not guarantee effective use of mosquito nets [23]. The usage rate for children is less than 35%. Our results differ from those of Ladu *et al.* [15]. This discrepancy may be due to the data source: our data are primary, whereas Ladu et al.’s data are from the 2011–2012 Demographic and Health Surveys.

The ownership and usage rates of LLINs differ slightly between the two study areas. Ownership is higher in Man than in Korhogo (94% versus 80%), while usage is higher in Korhogo than in Man (91% versus 77%). These differences are statistically significant at the 5% threshold, though moderate in magnitude.

The low level of transmission observed in Korhogo compared to Man by Adja *et al.* [2] could be partly due to the high rate of effective LLIN use by households in Korhogo, unlike in Man, where the population owns more LLINs but uses them less. Our results are consistent with those observed in three health districts in Cameroon [6]. In health districts with different levels of malaria prevalence, populations had different ownership and use rates from one district to another.

One of the main reasons cited for not using LLINs in Kaforo is discomfort due to heat. This finding is consistent with previous studies that emphasized behavioral and contextual barriers as major obstacles to optimal use of LLINs [24]. These studies conclude that the reasons for non-use of LLINs in malaria-endemic countries are human factors (sociocultural beliefs and practices), factors related to mosquito nets and housing structure, and access to mosquito nets. Socioeconomic and educational factors also influence LLIN ownership and use. Several studies have shown that education level is positively correlated with the adoption of preventive behaviors against malaria [7]. The higher school enrollment rate in Man (66%) than in Korhogo (34%) could explain the higher LLIN ownership there. Nevertheless, a significant proportion of respondents in Camp Sea (46%) and Logokaha (38%) could not explain why they did not use LLINs. A similar situation was observed in the Dimbokro health district, where 19.8% of households could not explain why they did not use LLINs [25].

The use of other prevention methods, such as insecticide coils and sprays and window nets, particularly in Camp Sea, shows that people are employing multiple strategies. This could be related to the behavior of malaria vectors. The main malaria vectors in Côte d’Ivoire (*An. gambiae* and *An. funestus*) bite both inside and outside homes, as well as during times when people are not necessarily asleep [3,12,16,18].

Low rates of respondents citing sanitation as a control method (ranging from 2% to 18% across our sites) suggest that environmental management measures (e.g., drainage, standing water removal, living environment hygiene, waste management) are less recognized as anti-malaria strategies than individual measures (e.g., mosquito nets, insecticides). This hierarchy of responses is consistent with numerous surveys in which “direct” practices (e.g. LLINs, spraying, and repellents) dominate community perception while sanitation appears to be a diffuse measure. It is often considered more the responsibility of communities, municipalities, or health structures than households and requires a technical understanding of larval habitats and vector ecology [24].

The low mention of sanitation may also reflect a disconnect between knowledge and action. Communities that are aware that the environment plays a role in malaria control may not prioritize it as an effective control method. A lack of material or organizational resources to take action is another reason. Studies on larval habitat management have shown that although environmental practices exist, communities adopt them heavily depending on resources, collective coordination, and institutional support [25].

The representativeness of our sites may be limited. For example, if the health district includes rural and urban areas, selecting one site per environment may not adequately represent the different areas. Another limitation is the lack of basic information on LLINs (e.g. number, insecticide used) in our various study sites.

Although the study was conducted after the national mass distribution of LLINs, it is difficult to attribute any reduction in malaria transmission solely to possession or use of these mosquito nets. The behavior regarding LLIN ownership and use observed among respondents should be associated with an assessment of disease prevalence. This would allow for a better evaluation of how acceptability and use of this protective measure affects the disease. The timing of the survey may influence some of the results. Although the dry season is reported to be the best time for this type of study, year-round studies would provide a more comprehensive assessment.

## Conclusion

The populations of the sentinel sites in Korhogo and Man are well aware of how malaria is transmitted and its symptoms. LLIN is the most widely used preventive tool among these populations, often used alongside other preventive measures, such as insecticide coils, window curtains or nets. However, our analysis shows that coverage is incomplete and does not comply with WHO and NMCP recommendations. LLIN ownership and use rates appear relatively consistent with NMCP expectations but are impacted by obstacles such as heat and suffocation, affecting access to and use of LLINs. These obstacles highlight the need to improve understanding of the issues related to the non-use of this essential means of prevention and to continue raising awareness among populations by adapting the approach to the local context. Conducting such surveys in other NMCP sentinel sites also seems important.

## Acknowledgements

We would like to thank the administrative and traditional authorities, as well as the people interviewed in Korhogo and Man, who gave their time to participate in the survey.

## Conflict of interest, funding, and ethical principles

The authors declare that this research did not receive any financial support and that they have no conflict of interest. The study was approved by the health authorities in each locality. Permission to conduct the survey was obtained from neighbor-hood/village leaders. In addition, verbal consent was obtained from respondents prior to their participation in the study.

## Authors’ contributions

KFA, AMA, and FF participated in developing the topic and writing the manuscript. KFA and KRMA participated in the survey. KFA conducted data entry and analysis. KFA, KRMA, and FF participated in writing the manuscript. KFA, KRMA, AMA, and FF participated in editing the manuscript, and all authors agreed to its publication.
